# Personality Traits and Perceived Food Allergy: A Pilot Exploratory Study

**DOI:** 10.3390/jcm15093185

**Published:** 2026-04-22

**Authors:** Gianluca Pandolfo, Clara Lombardo, Carmela Mento, Mattia Porcino, Raffaele Cordiano, Vincenzo Papa, Federica Vita, Giuliana Amato, Fabrizio Turiaco, Sebastiano Gangemi, Paola Lucia Minciullo

**Affiliations:** 1 Department of Biomedical and Dental Sciences, Morphological and Functional Images, University of Messina, 98125 Messina, Italy; gpandolfo@unime.it (G.P.); porcino.mattia@gmail.com (M.P.);; 2 Psychiatry Unit, Polyclinic Hospital, University of Messina, 98125 Messina, Italy; 3 Department “Scienze della Salute”, University of Magna Graecia, 88100 Catanzaro, Italy; 4 Department of Clinical and Experimental Medicine, University of Messina, 98125 Messina, Italyfederica.vita75@gmail.com (F.V.); giulianaamato89@hotmail.it (G.A.); sebastiano.gangemi@unime.it (S.G.); paolalucia.minciullo@unime.it (P.L.M.); 5 School and Unit of Allergy and Clinical Immunology, University of Messina, 98125 Messina, Italy

**Keywords:** food allergy, maladaptive personality traits, psychosomatic factors, quality of life, integrative medicine, PID-5

## Abstract

**Background**: A substantial discrepancy exists between the prevalence of perceived food allergy and that of food allergy confirmed by objective diagnostic methods. The misperceptions of food-related symptoms in the absence of a confirmed food allergy may be related to maladaptive personality traits, such as neuroticism, which may shape symptom perception, illness experience, and the therapeutic alliance. From an integrative medicine perspective, maladaptive personality traits may be associated with the reporting and interpretation of food-related symptoms. **Objective:** This pilot exploratory study examined associations between personality traits and perceived food allergy. **Methods:** Twenty-one adults reporting food allergy symptoms underwent allergological assessment and standardized psychological evaluation. Participants were classified as having confirmed (*n* = 10) or excluded (*n* = 11) immune-mediated food allergy. Maladaptive personality traits were assessed using the Personality Inventory for DSM-5 Brief Form (PID-5-BF), and quality of life was measured using the WHOQOL-BREF. Between-group comparisons were performed using Mann–Whitney U tests. **Results:** Patients without confirmed food allergy demonstrated significantly higher Antagonism scores compared with those with confirmed allergy (*p* = 0.003; g = 1.03). No significant differences emerged in other personality domains, although moderate-to-large effect sizes were observed for Negative Affectivity, Disinhibition, and Psychoticism. These findings should be interpreted with caution given the small sample size and the exploratory nature of the study and should be considered preliminary and hypothesis-generating. **Conclusions:** Patients with confirmed food allergy may be primarily burdened by environmental limitations associated with disease management, whereas individuals without confirmed allergy may exhibit higher levels of maladaptive personality traits, particularly antagonism. Integrating psychological assessment into allergy care may support patient-centered, evidence-based integrative approaches in conditions marked by diagnostic uncertainty.

## 1. Introduction

Personality is generally defined as a set of enduring characteristics that differentiate individuals and reflect a stable organization of behavioural patterns. Personality traits represent relatively stable dimensions of thoughts, feelings, and behaviours that shape individual functioning. They influence how people experience, interpret, and cope with stress, thereby shaping whether stress remains a psychological phenomenon or evolves into physical illness, requiring integrative therapeutic approaches that address both mind and body [1]. Adverse reactions to food are frequently self-reported in clinical settings; however, self-reported food allergy does not necessarily correspond to immune-mediated food allergy confirmed by allergological assessment. In many cases, patients may attribute a wide range of symptoms to food intake in the absence of objective evidence of an IgE-mediated or other immune-mediated mechanism. This discrepancy is clinically relevant and may reflect the coexistence of different conditions, including confirmed food allergy, non-allergic adverse food reactions, medically unexplained symptoms, and psychosomatic symptom presentations. Although these conditions may overlap at the phenomenological level, they are conceptually distinct and should not be considered interchangeable.

Increasing evidence shows that psychosocial variables, including coping mechanisms, exposure to stressful events, and personality types, contribute to the development of a broad spectrum of psychosomatic manifestations. In particular, maladaptive personality traits have been associated with various psychosomatic symptoms, including chronic pain [2,3], fatigue, headache [4], and gastrointestinal disturbances [5]. Associations with self-reported food allergy have also been reported, although findings remain controversial. Interestingly, a substantial discrepancy exists between the prevalence of perceived food allergy and that of food allergy confirmed by objective diagnostic methods. In some cases, the misinterpretation of food-related symptoms may be influenced by psychosomatic mechanisms or psychological factors; however, these processes should not be considered equivalent to confirmed immune-mediated food allergy. Some authors suggested that neuroticism may be a trait associated with perceived food allergy in both sexes; moreover, individuals who self-report food allergy tend to experience higher levels of anxiety and poorer mental health outcomes [6]. However, evidence is mixed: in one study [7], self-reported food allergy was associated with greater anxiety and poorer self-reported mental health in adolescents, but not in adults.

In this context, dimensional models of personality provide a useful perspective for examining maladaptive personality traits associated with the expression of somatic symptoms. The dimensional model of personality proposed in DSM-5 [8] allows for the assessment of maladaptive traits through the Personality Inventory for DSM-5 (PID-5), which assesses personality functioning in five broad domains [9]. Specifically, the domains of negative affectivity, which include anxiety, emotional lability, and hostility; detachment, characterised by social withdrawal and reduced capacity for pleasure; disinhibition, associated with impulsivity and poor behavioural regulation; and psychoticism, involving eccentric or disorganised patterns of thinking and perception, have been identified as relevant dimensions in relation to psychological vulnerability. These areas of maladaptive personality may contribute to the subjective experience and persistence of physical symptoms, particularly in clinical conditions characterised by the absence of objective organic findings.

Within this complex interplay, it remains unclear whether dysfunctions in personality domains contribute causally to perceived food allergy symptoms, or whether both arise from shared underlying mechanisms. This study was designed as a pilot exploratory investigation aimed at generating preliminary, hypothesis-generating evidence, given the limited sample size and the absence of prior data in this specific area. The aim of the present study was to investigate the relationship between psychosomatic parameters, personality traits, and perceived food allergy.

## 2. Materials and Methods

### 2.1. Participants

All patients were assessed at the allergy clinic of a tertiary university hospital in Southern Italy for suspected food allergy. The sample consisted of 21 adult participants, 18 females and 3 males, aged between 19 and 65 years (mean age 39.1 ± 12.7 years). At presentation, patients reported symptoms perceived as food allergy, including erythema, cutaneous and/or pharyngeal pruritus, urticaria, angioedema, dyspnoea, abdominal pain, diarrhoea, and loss of consciousness. The time interval between symptom onset and allergy assessment varied considerably, ranging from 7 to 139 months. The mean age at diagnosis was 36.3 years (range: 18–48 years).

Each participant provided informed consent after receiving detailed information about the study procedures.

### 2.2. Inclusion and Exclusion Criteria

Inclusion criteria were age ≥ 18 years and patients attending their first allergy consultation following a referral by their General Practitioner due to a suspected food allergy to be confirmed or ruled out through allergy assessment.

Exclusion criteria included age < 18 years, presence of severe psychiatric conditions interfering with study participation, and refusal or inability to provide informed consent.

### 2.3. Procedure

Each patient underwent a thorough allergy interview, subsequently followed by the administration of the following standardized psychometric instruments.

The Personality Inventory for DSM-5–Brief Form (PID-5-BF) [10] is a self-administered instrument composed of 25 items designed to evaluate five major maladaptive personality domains: Negative Affectivity, Detachment, Antagonism, Disinhibition, and Psychoticism. These domains reflect characteristic patterns of emotional experience, behavioural regulation, and interpersonal functioning. Items are rated on a four-point Likert ranging from 0 (very false or often false) to 3 (very true or often true). Previous research has demonstrated good longitudinal stability of the personality traits assessed by the PID-5 [11].

The WHOQOL-BREF is a self-administered questionnaire consisting of 24 items, structured into four main domains: physical health, psychological health, social relationships, and environment. Two additional items assess individuals’ overall perception of quality of life and general health status [12,13]. Scores may be calculated using different scoring procedures, including raw scores and transformed scales ranging from 0–20 or 0–100. For the purposes of the present study, all results were expressed using the 0–100 scale, where higher scores indicate better perceived quality of life. On this scale, a score of 0 represents extremely poor quality of life, whereas a score of 100 reflects excellent quality of life. A value of 50 may be considered indicative of an intermediate condition, neither positive nor negative [14]. The WHOQOL-BREF was developed for application across diverse cultural contexts and has demonstrated satisfactory psychometric properties both in cross-cultural research and in comparisons among subgroups within the same population [14,15].

### 2.4. Statistical Analysis

Descriptive and inferential statistical analyses were performed. Given the small sample size, group comparisons were conducted using the Mann–Whitney U test. Normality was assessed using the Shapiro–Wilk test. Effect sizes were calculated using Hedges’ g to allow standardized interpretation of group differences and comparability with previous literature. Results with *p* < 0.05 were considered significant. Statistical analyses were performed using SPSS 25.0 (SPSS Inc., Chicago, IL, USA).

## 3. Results

### 3.1. Allergy Findings

Participants were divided into two subgroups based on the outcome of allergy investigations, according to clinical history, in vivo and in vitro tests. Group A was composed of 11 patients (1 male, 10 females; Mean Age: 40.82 ± 12.68) with excluded food allergy and group B comprised 10 patients (2 males, 8 females; Mean Age: 32.70 ± 9.17) with confirmed food allergy.

Regarding allergy pathway, 10 participants (47.6%) received a confirmed diagnosis of food allergy based on a highly evocative clinical history, positive skin prick or prick-by-prick testing for food allergens, and/or elevated serum-specific IgE levels to food allergens. The remaining 11 participants (52.4%) did not meet diagnostic criteria for immune-mediated food allergy, due to a clinical history weakly suggestive of allergic reactions and sometimes negative allergy tests.

Comparative analyses between Group A and Group B focused on maladaptive personality traits and perceived quality of life, as described below.

### 3.2. Maladaptive Personality Traits

Comparisons between participants with confirmed food allergy and those in whom allergy was excluded were performed using the Mann–Whitney U test. Descriptive statistics and effect size estimates are reported in Table 1. A statistically significant between-group difference emerged for the Antagonism domain, with higher scores observed in participants without confirmed food allergy (*p* = 0.003). The magnitude of this difference was large (Hedges’ g = 1.03).

No statistically significant differences were observed for Negative Affectivity (*p* = 0.085), Detachment (*p* = 0.349), Disinhibition (*p* = 0.072), or Psychoticism (*p* = 0.114). Effect size estimates suggested possible differences; however, given the lack of statistical significance and the very small sample size, these estimates are likely to be unstable and should not be interpreted as indicative of true underlying effects, particularly in the absence of statistical significance.

### 3.3. Quality of Life

Group comparisons between participants with confirmed and excluded food allergy did not reveal statistically significant differences across WHOQOL-BREF domains (all *p* > 0.05). Effect size estimates suggested potential differences across domains; however, none of these differences reached statistical significance and should not be interpreted as evidence of meaningful group differences. Small effect sizes were observed for the physical health (Hedges’ g = −0.24) and psychological health domains (g = 0.18). A moderate effect size emerged for social relationships (g = −0.50), with higher scores reported by participants with confirmed food allergy. Notably, a moderate-to-large effect size was observed for the environmental domain; however, this difference did not reach statistical significance (Table 2).

## 4. Discussion

The present pilot exploratory study examined maladaptive personality traits and quality of life in patients self-reporting food allergy. Given the small sample size, the findings should be interpreted with caution and considered preliminary. Despite comparable symptom presentations, meaningful psychological differences emerged between groups, underscoring the complexity of psychological and immunological interplay in this population.

The most robust finding concerned the Antagonism domain of the PID-5, which was significantly higher in patients without confirmed food allergy and showed a large effect size. Within the DSM-5 Alternative Model of Personality Disorders, antagonism reflects maladaptive interpersonal functioning characterized by hostility, mistrust, and oppositional attitudes [16]. Previous research has suggested that antagonistic traits may be associated with difficulties in interpersonal relationships and engagement with healthcare providers [17]. However, these associations were not directly assessed in the present study and should therefore be interpreted cautiously. In this context, higher levels of antagonism may be associated with differences in symptom perception and reporting; however, no inference regarding directionality or causality can be made based on the present cross-sectional data. This interpretation remains hypothetical and requires confirmation in larger and methodologically more robust studies.

In allergy settings, the absence of objective confirmation may intensify patient dissatisfaction and complicate communication. Difficulties in accepting negative diagnostic results have been widely described in patients with medically unexplained symptoms and somatic distress [18,19]. Somatic symptom disorder (SSD) is a DSM-5 diagnosis involving one or more distressing physical symptoms accompanied by excessive thoughts, feelings, or behaviours related to the symptoms. Previous research has suggested associations between severe somatization and maladaptive personality traits.

In this study design, causal relationships cannot be established; however, high levels of antagonistic traits may be related to differences in illness behavior, expectations regarding diagnostic procedures, and the interpretation of bodily sensations, rather than indicating the diagnostic outcome itself. Antagonistic traits may be related to differences in the perception and interpretation of food-related symptoms; however, this relationship remains speculative and cannot be interpreted as causal. These interpretations should also be evaluated in the context of the potential diagnostic uncertainty associated with the lack of a systematic oral food challenge. Negative affectivity is associated with increased symptom vigilance and emotional reactivity to bodily sensations [20]. In contrast, disinhibition and psychoticism traits may lead to impulsive health-related behaviors and idiosyncratic symptom interpretation [21]. It is important to acknowledge that affective symptoms, including anxiety, depression, and stress, which were not evaluated in the current study, may have impacted these results. In our cohort, moderate to large effect sizes were observed for these domains; however, these differences did not achieve statistical significance and should be interpreted with caution. In small samples, effect size estimates may be unstable and susceptible to random variation and therefore should be regarded as exploratory rather than indicative of significant associations.

In terms of quality of life, there were no statistically significant differences between groups in the WHOQOL-BREF domains. Effect size estimates indicated possible differences, especially in the environmental domain; however, these results did not achieve statistical significance and should be interpreted cautiously. Instead of demonstrating a validated difference, this pattern may reflect random variation within a small sample and therefore cannot be interpreted as indicative of a consistent difference, warranting further investigation in larger studies. Previous studies indicate that food allergy can create significant challenges associated with dietary restrictions, perpetual vigilance, anxiety regarding inadvertent exposure, and constraints in social and professional environments [22,23,24,25]. Nevertheless, the current findings do not furnish adequate evidence to substantiate these differences in this sample. Conversely, psychological and physical quality-of-life domains appeared largely comparable between groups. Prior studies have shown that perceived symptom severity, illness uncertainty, and health-related anxiety often exert a stronger influence on quality of life than objective disease markers [26]. Our findings support this interpretation, suggesting that both confirmed allergy and diagnostic uncertainty may represent distinct but equally distressing clinical conditions. Given the limited statistical power, the study is primarily hypothesis-generating, and the findings should be interpreted as exploratory rather than confirmatory.

From a clinical perspective, integrating psychological screening into allergy diagnostic pathways may facilitate more effective communication and reduce frustration in cases of diagnostic exclusion. Such an approach is consistent with current recommendations advocating for multidisciplinary and biopsychosocial models of care in chronic medical conditions [27].

## 5. Limitations

Several limitations should be acknowledged. First, the study was monocentric and involved a small sample size, limiting statistical power and generalizability. Second, the cross-sectional design prevents causal inference regarding the directionality of associations between psychological traits and diagnostic outcome. Third, all psychological measures were self-reported, which may introduce response bias and shared method variance. Fourth, psychiatric diagnoses and additional relevant constructs, such as depression, health anxiety, or somatic symptom burden, were not systematically assessed. In particular, depressive, anxiety, and stress-related symptoms were not specifically assessed. These factors may influence both symptom perception and personality trait expression and could therefore represent potential confounders in the interpretation of the present findings. Given the pilot nature of the study, these findings should be considered preliminary and primarily hypothesis-generating. Overall, the small sample size substantially limits statistical power and the stability of the estimates, and the results should therefore be interpreted as exploratory and hypothesis-generating rather than confirmatory. In addition, the cross-sectional design precludes any inference regarding temporal or causal relationships between personality traits and perceived food-related symptoms. The observed associations may reflect bidirectional or confounded relationships, and longitudinal studies are required to clarify these dynamics. Finally, effect size estimates may be unstable in small samples and should therefore be interpreted with caution.

## 6. Conclusions

In our cohort, 52% of individuals who believe themselves to have a food allergy have no objective supporting evidence. Maladaptive traits have been hypothesized to be associated with the perception of food-related symptoms; however, the present findings provide only preliminary and exploratory indications that require confirmation. The present findings provide preliminary and exploratory indications that maladaptive personality traits, particularly antagonism, may be tentatively associated with self-reported food allergy in this sample. However, these results require confirmation in larger and methodologically more robust studies. Antagonistic personality traits may be associated with differences in the interpretation of symptoms and in the patient–clinician interaction, although these aspects were not directly assessed in the present study. Therefore, it is important to promote the assessment and a clearer definition of the role of psychosocial factors and to implement integrated interventions that include psychological therapies for the treatment of these conditions. Finally, these findings underscore the significance of a comprehensive evaluation that incorporates both medical and psychological perspectives in allergy practice.

## Figures and Tables

**Table 1:** Group comparisons were performed using the Mann–Whitney U test. Test statistics (U), *p*-values, and effect sizes (Hedges’ g) are reported.

PID-5 Domain	Group A (*n* = 11) M ± SD	Group B (*n* = 10) M ± SD	Mann–Whitney U	*p*-Value	Effect Size (Hedges’ g)
Negative Affectivity	1.02 ± 0.34	0.72 ± 0.52	30.5	0.085	0.66
Detachment	0.74 ± 0.44	0.55 ± 0.21	41.5	0.349	0.53
Antagonism	0.47 ± 0.37	0.14 ± 0.22	14	0.003	1.03
Disinhibition	0.88 ± 0.45	0.56 ± 0.51	29	0.072	0.64
Psychoticism	0.70 ± 0.50	0.34 ± 0.32	32.5	0.114	0.82

**Table 2:** Group comparisons were performed using the Mann–Whitney U test. Test statistics (U), *p*-values, and effect sizes (Hedges’ g) are reported.

WHOQOL-BREF Domain	Group A (*n* = 11) M ± SD	Group B (*n* = 10) M ± SD	Mann–Whitney U	*p*-Value	Effect Size (Hedges’ g)
Physical Health	68.45 ± 34.41	76.70 ± 31.89	40	0.282	−0.24
Psychological Health	51.73 ± 37.03	44.90 ± 34.60	51	0.776	0.18
Social Relationships	41.09 ± 12.66	46.40 ± 6.59	42	0.355	−0.50
Environmental	64.82 ± 37.06	37.40 ± 37.27	35.5	0.168	0.71

## Data Availability

The data presented in this study are available upon request from the authors who reserve the right to make them available.
